# Emolabeling Effectively Reduces the Influence of Ambiguous Labeling on Food Packages Among Grocery Store Shoppers

**DOI:** 10.5539/gjhs.v7n4p12

**Published:** 2014-12-16

**Authors:** Gregory J. Privitera, Caitlin J. Brown, James J. Gillespie

**Affiliations:** 1 Department of Psychology, St. Bonaventure University, St. Bonaventure, NY, USA; 2 Healthcare Researcher and Consultant, Chicago, IL, USA

**Keywords:** nutrition label, emoticons, emolabeling, health, food policy

## Abstract

Despite increased regulations and policy enforcement for nutrition labeling, ambiguous labels on food items can still have deleterious effects on consumer perceptions of health. The present study used a counterbalanced within-subjects design to test if *emolabeling*—the use of emoticons to convey health information (happy = healthy; sad = not healthy)—will reduce the effects of ambiguous labels on consumer perceptions of the healthfulness of a food item. 85 grocery store shoppers were shown nutrition labels for a low calorie (LC) and a high calorie (HC) food with/without emolabels, and with an ambiguous label that either implied the food was healthy or unhealthy. Results showed that emolabels reduced the effectiveness of ambiguous labels: consumers rated the LC food as healthier and the HC food as less healthy when emolabels were added. The results suggest that, if implemented, this image-based emolabeling system could possibly be an effective buffer against the use of ambiguous labeling by food manufacturers.

## 1. Introduction

Obesity is a public health, agricultural, and economic concern (Finucane et al., 2011; Kochanek, Xu, Murphy, Miniño, & Kung, 2009). U.S. agriculture policy includes incentives that can lead to overproduction and low prices for sweet and fat laden foods that likely contributes to obesity in the United States and the healthcare costs associated with it (Cawley & Meyerhoefer, 2008; O’Grady & Capretta, 2012; Wallinga, 2010). In terms of food choice, nutrition ranks as the second most influential factor influencing food purchases behind only taste (Guthrie, Derby, & Levy, 1999). A key policy effort to regulate the labeling of nutrition information on food packages is the Nutrition Labeling and Education Act (NLEA), which was passed by Congress in 1990 to regulate nutrition labeling and disclosure (NLEA, 1990). One key aim of the NLEA was to safeguard consumers from inaccurate or misleading health-related claims on packages, in part because such claims can substantially impact food purchasing.

One persistent concern is the use of ambiguous labeling on food packages in which a label is technically accurate but can potentially misinform consumers (Pomeranz, 2011; Prevention Institute, 2002). For example, terms such as “lite/light” (in terms of weight or color; not health) are often used in an ambiguous manner. Manufacturers also can highlight one healthy nutrient such as “whole grains” in a food that is otherwise high fat/high sugar (Privitera, 2008; Silverglade, 1996). Recently, the Food and Drug Administration (FDA) authorized actions to investigate possible misleading labeling in response to increased consumer complaints that package labels misrepresented the actual health benefits of certain foods (FDA, 2009). However, one constraint of initiatives for stronger enforcement and increased industry regulation is First Amendment protections of “commercial speech” by which manufacturers can challenge increased regulations in court (Rubin v. Coors Brewing Co., 1995; Zauderer v. Offıce of Disciplinary Counsel of the Supreme Court of Ohio, 1985). Some ambiguous labels, such as the examples given earlier, are technically accurate and therefore can fall under the category of “commercial speech.” Thus, many ambiguous labels, if technically accurate, will likely remain on food packages even if such labels could potentially lead consumers to misconstrue the actual healthfulness of a food item. It would therefore be advantageous to develop a labeling strategy to counteract the possible deleterious effects of ambiguous labels on consumer perceptions of health for food labeled items.

Evidence shows that consumers tend to preferentially evaluate prominently positioned nutrition information, with fat content, sugar content, and calories per serving viewed most (Graham & Jeffery, 2011). Greater attention is also given to nutrition information in more prominent display sizes, color schemes, and locations on the package, such as front-of-package labeling (Bialkova & van Trijp, 2010)—with enhanced healthy product choices even when viewing time constraints are added (van Herpen & van Trijp, 2011). Picture-based presentation of nutrition information can further enhance time spent viewing nutrition labels (Pennings, Striano, & Oliverio, 2013), consistent with a recent review in which researchers suggested that simplifying nutrition label presentation can positively influence the informativeness of nutrition labels and can enhance healthy food choice (Graham, Orquin, & Visschers, 2012).

In the present study, we tested the utility of an image-based nutrition labeling system called, *emolabeling* (Privitera, Vogel, & Antonelli, 2013). Using emolabeling, emoticons are used to represent emotional correlates of health (happy = healthy; sad = not healthy). It is a potentially robust labeling strategy in that it uses emoticon images to simplify nutrition labeling and has been shown to effectively improve health knowledge and healthy food choices even among children who cannot read and write (Privitera et al., 2013; Privitera, Phillips, Misenheimer, & Paque, 2014), and thus should effectively improve health knowledge among consumers at all health literacy levels. Also, emolabels are easy-to-recognize images that intuitively correlate with health and can bring greater attention to health information (Privitera et al., 2014). Therefore, we tested the utility of emolabeling on nutrition labels by recording the healthfulness ratings of consumers for a low-calorie (LC) and high-calorie (HC) food with/without emolabels present, and with ambiguous labels that implied that a food was either healthy or unhealthy.

## 2. Method

### 2.1 Participants

A sample of 85 grocery store shoppers at a local supermarket in Western New York was asked to participate in a survey. Of the 85 grocery store shoppers sampled, 41 were men, 44 were women, mean/SD age was 53.1±16.4 (years), height was 169.9.9±12.2 (cm), and weight was 80.5±18.8 (kg). BMI scores ranged from 19.6 to 36.2 with a mean/SD of 27.7±4.6 (kg/m^2^). All participants were grocery store shoppers who volunteered to complete the study. 78% of respondents indicated being the primary shopper and all participants reported being familiar with reading nutrition labels in a grocery store setting.

### 2.2 Sampling Procedures

A systematic sampling procedure was used in which every fifth shopper was asked to participate. This strategy ensured that a consistent sampling procedure was employed throughout the day to manage the observational period. Shoppers were asked to complete a brief survey to “determine how to serve them better.” The response rate for requests to participate was 77% (85 out of 111 requests), which is a satisfactory rate for selecting representative samples (Baruch, 1999; Privitera, 2014).

### 2.3 Procedures

The study was conducted inside a local grocery store in the Western New York area on site over the course of one week during daytime hours. A table with chairs was set up inside the store at an exit point where shoppers had to travel in order to leave the store. Participants were read and signed an informed consent, then completed a brief survey and a demographic questionnaire. The survey consisted of a total of 8 nutrition labels. Four labels were for a healthy low calorie (LC) spring mix lettuce, which was 15 calories. The other four labels were for a less healthy high calorie (HC) chocolate peanut butter pie, which was 600 calories. The two food items chosen here we chosen because these discernibly fall into a healthy versus unhealthy category, although at present it is too preliminary to state exact criteria for the use of emolabels for any food type—such work is currently being completed to identify possible criteria. To manipulate emolabels and ambiguous labeling, a 2 × 2 design was used with the LC and with the HC food in which emolabels were present/absent, and an ambiguous label was added to imply that the food item was healthy/unhealthy.

Emolabels were used to specifically label the sugar and fat content. Fat and sugar were chosen for emolabeling because consumers most often attend to these nutrients in nutrition labels (Graham & Jeffery, 2011), and when consumed at low levels, are regarded as part of a healthy diet—based on dietary guidelines provided by the U.S. Department of Health and Human Services (HHS, 2010). To implement emolabeling, a happy face emoticon was used to indicate “healthy;” a sad face emoticon was used to indicate “unhealthy.” At the table where participants completed the survey, a small card was placed showing an image of each emoticon and whether it indicated healthy or unhealthy in each nutrition label. The added ambiguous labels were “Wholesome Goodness” and “Nutritionally Deplete.” These labels were matched as close as possible on word count (both were two words) and character length. “Nutritionally Deplete” was the ambiguous label that implied a food was unhealthy; “Wholesome Goodness” was the ambiguous label that implied a food was healthy.

**Figure 1:**
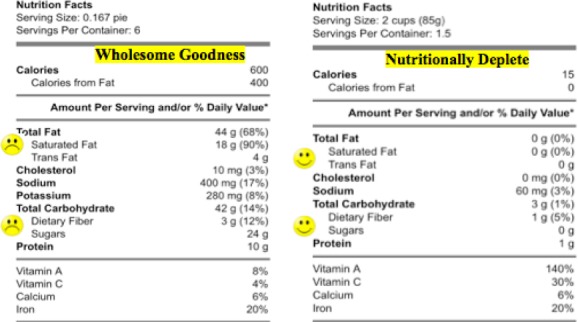
A sample image of a nutrition label for the LC nutrition label (right image) and HC nutrition label (left image) with an ambiguous label and the emolabels for sugar and fat added. In total, 4 LC and 4 HC food nutrition labels were shown and rated

In total, there were 8 labels rated: 4 LC and 4 HC food nutrition labels were shown to consumers that varied by emolabeling (present, absent) and ambiguous label type (implied healthy, implied unhealthy). Thus, the LC food nutrition label had “Nutritionally Deplete” on the label with and without emolabels added, and the LC nutrition label had “Wholesome Goodness” on the label with and without emolabels added. Likewise, the HC nutrition label had “Nutritionally Deplete” on a label with and without emolabels added, and the HC nutrition label had “Wholesome Goodness” on a label with and without emolabels added. Figure 1 shows an example of two nutrition labels in the survey for which participants rated healthfulness: one for the LC and one for the HC food with an ambiguous label and the emolabels added.

For each nutrition label (8 total labels), participants rated the healthfulness of the food on a 5-point scale from 1 (Very Unhealthy) to 5 (Very Healthy). The order of the nutrition labels in the survey was counterbalanced using a Latin square to minimize order effects. Once participants completed all materials, they were debriefed, thanked for their time, and dismissed. The SBU institutional ethics committee approved the study procedures.

### 2.4 Statistical Analysis

A 2 × 2 within subject’s factorial ANOVA was used with emolabels (present, absent), and ambiguous label type (implied food was healthy, implied food was not healthy) as within-subjects factors. Healthfulness ratings were the dependent variable. A separate 2 × 2 ANOVA was computed for the LC labels, and for the HC labels. Sex was initially included, but later removed as a factor because it showed no significance in analyses reported here. Planned related samples *t* tests were computed to test interactions with a Bonferroni procedure used to control for experimentwise alpha. All tests were conducted at a .05 level of significance.

## 3. Results

**Figure 2:**
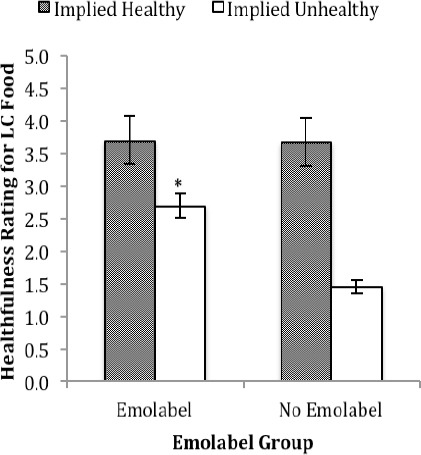
Healthfulness ratings for the LC nutrition label. An asterisk indicates significance between the groups in which the ambiguous label implied that the LC food was unhealthy at *p* < .001

For the LC food, an analysis showed a main effect of emolabeling, F(1, 84) = 30.92, *p* < .001 (*R^2^* = .27), with higher ratings (*M*±SD) of healthfulness with (3.2±1.4) versus without (2.6±0.9) emolabels. Also evident was a main effect of ambiguous label type, F(1, 84) = 121.48, *p* < .001 (*R^2^* = .59), with higher healthfulness ratings for the food with an ambiguous label that implied the food was healthy (3.7±1.0) versus unhealthy (2.1±1.3). An emolabeling × ambiguous label type interaction was also evident, F(1, 84) = 26.54, *p* < .001 (*R^2^* = .24). As shown in Figure 2, adding emolabels (two happy faces) significantly increased healthfulness ratings when the ambiguous label implied that the LC food was not healthy, *t*(84) = 6.07, *p* < .001, *d* = 0.66.

**Figure 3:**
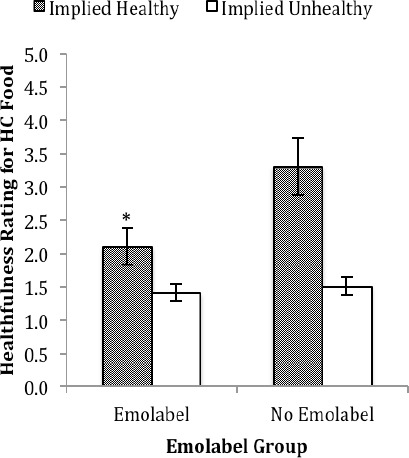
Healthfulness ratings for the HC nutrition label. An asterisk indicates significance between the groups in which the ambiguous label implied that the HC food was healthy at *p* < .001

For the HC food, an analysis showed a main effect of emolabeling, F(1, 84) = 30.69, *p* < .001 (*R^2^* = .27), with lower healthfulness ratings with (1.7±1.2) versus without (2.4±1.1) emolabels. Also evident was a main effect of ambiguous label type, F(1, 84) = 86.02, *p* < .001 (*R^2^* = .51), with lower healthfulness ratings for the food with an ambiguous label that implied the food was unhealthy (1.5±0.9) versus healthy (2.7±1.3). An emolabeling × ambiguous label type interaction was also evident, F(1, 84) = 27.12, *p* < .001 (*R^2^* = .24). As shown in Figure 3, adding emolabels (two sad faces) significantly reduced healthfulness ratings when the ambiguous label implied that the HC food was healthy, *t*(84) = -5.97, *p* < .001, *d* = -0.65.

## 4. Discussion

The utility of emolabeling to reduce the influence of ambiguous labels on consumer perceptions of health was tested. The results show a clear pattern in support for the hypothesis tested with emolabels added to inform consumers about the healthfulness of the sugar and fat content in the food. With an ambiguous label on a nutrition label, adding emolabels to clarify the healthfulness of the sugar and fat content improved the accuracy of health ratings: participants rated the LC food as healthier when emolabels were added, and rated the HC food as less healthy when emolabels were added. Thus, emolabeling improved the accuracy of health ratings for the LC and the HC food even when ambiguous labels incorrectly implied that a food was healthy/unhealthy.

An important implication of the results here is that emolabeling improved the informativeness of nutrition labels. Such an image-based strategy is a potentially practical strategy to implement. Emolabeling is effective at communicating health across all health literacy levels, even among those with low/no literacy (Privitera et al., 2013, 2014); and it uses simple expressions of emotion (using emoticons) that are recognized universally (Darwin, 1979) to communicate correlates of health (happy = healthy, sad = not healthy). Emolabeling can therefore enhance the informativeness of nutrition information, as demonstrated in this study, and appears to be a dynamic strategy with the potential for widespread application across multiple ecological contexts.

Some limitations for this study can be identified here. First, only two types of ambiguous labels were tested. Whether other types of ambiguous labels (such as labels with different phrases, word counts, or character lengths) could have effectively reduced the effectiveness of emolabels cannot be determined here. Future studies can certainly test this possibility to identify the ecological validity of the results reported here. Second, we chose only two food types, one that was very low calorie, and one that was very high calorie. The utility of emolabeling when “health” is more ambiguous (such as for foods with more moderate calorie counts) cannot be addressed here, and would likely require a more collaborative effort to identify such criteria. Finally, although the power for this study was more than satisfactory (i.e., for all tests, observed power was at least .99) (Privitera, 2015), it would be advantageous to conduct this type of study with larger samples and in other food venues, other than a grocery store setting, to strengthen the population validity and generalizability of the results reported here for the present study.

Overall, the results in the present study provide direct evidence showing that emolabeling on nutrition labels can effectively reduce the possible deleterious influence of ambiguous labeling of food packages. Future directions regarding emolabeling could create increased opportunities for identifying standard nutritional criteria for the use of emolabels, and voluntary adoption by industry as food manufacturers respond to increased societal demands for healthier foods. While food manufactures with unhealthy foods might be resistant to emolabeling, there is reason to think that those producing healthier foods could become advocates within the industry if nutritional criteria were established.

## References

[1] Baruch N. P (1999). Response rate in academic studies: A comparative analysis. Human Relations.

[2] Bialkova S, van Trijp H (2010). What determines consumer attention to nutrition labels?. Food Quality and Preference.

[3] Cawley J, Meyerhoefer C (2008). The medical care costs of obesity: An instrumental variables approach. J Health Econ.

[4] Darwin C (1979). The expression of emotion in man and animals.

[5] Finucane M. M, Stevens G. A, Cowan M. J, Danaei G, Lin J. K, Ezzati M (2011). National, regional, and global trends in body-mass index since 1980: Systematic analysis of health examination surveys and epidemiological studies with 960 country-years and 9.1 million participants. The Lancet.

[6] Food and Drug Administration (FDA) (2009). New front-of-package labeling initiative.

[7] Graham D. J, Jeffery R. W (2011). Location, location, location: Eye-tracking evidence that consumers preferentially view prominently positioned nutrition information. J Am Diet Assoc.

[8] Graham D. J, Orquin J. L, Visschers V (2012). Eye tracking and nutrition label use: A review of the literature and recommendations for label enhancement. Food Policy.

[9] Guthrie J. F, Derby B. M, Levy A. S, Frazao E (1999). What people know and do not know about nutrition. America’s eating habits: Changes and consequences.

[10] Kochanek K. D, Xu J, Murphy S. L, Miniño A. M, Kung H.-C (2009). Deaths: Final data for 2009. National Vital Statistics Reports.

[11] Nutrition Labeling and Education Act of 1990. Public Law 101-533, 104 Stat 2353.

[12] O’Grady M. J, Capretta J. C (2012). Assessing the economics of obesity and obesity interventions.

[13] Pennings M. C, Striano T, Oliverio S (2013). A picture tells a thousand words: Impact of an educational nutrition booklet on nutrition label gazing. Marketing Letters, August 28.

[14] Pomeranz J. L (2011). Front-of-Package Food and Beverage Labeling: New Directions for Research and Regulation. American Journal of Preventative Medicine.

[15] Prevention Institute Nutrition labeling regulations.

[16] Privitera G. J (2008). The psychological dieter: It’s not all about the calories.

[17] Privitera G. J (2014). Research Methods for the Behavioral Sciences.

[18] Privitera G. J, Phillips T. E, Misenheimer M. L, Paque R (2014). The effectiveness of “emolabeling” to promote healthy food choices in children preschool through 5th grade. International Journal of Child Health and Nutrition.

[19] Privitera G. J, Vogel S. I, Antonelli D. E (2013). Performance on a food health assessment using emoticons with pre-literacy-aged children. American Journal of Educational Research.

[20] Privitera G. J (2015). Statistics for the Behavioral Sciences.

[21] Rubin v (1995).

[22] Silverglade B (1996). The Nutrition Labeling and Education Act: A public health milestone is now under attack. Journal of Nutrition Education.

[23] U.S. Department of Health and Human Services (2010). Dietary Guidelines for Americans, 2010.

[24] U.S. Department of Health and Human Services (2011). Front-of-pack nutrition labels. Their effect on attention and choices when consumers have varying goals and time constraints. Appetite.

[25] Wallinga D (2010). Agricultural policy and childhood obesity: A food systems and public health commentary. Health Affairs.

[26] Zauderer v (1985). Office of Disciplinary Counsel of the Supreme Court of Ohio, 471 U.S.

